# Involvement of NMDA-AKT-mTOR Signaling in Rapid Antidepressant-Like Activity of Chaihu-jia-Longgu-Muli-tang on Olfactory Bulbectomized Mice

**DOI:** 10.3389/fphar.2018.01537

**Published:** 2019-01-09

**Authors:** Xing Wang, Zhilu Zou, Qinqin Shen, Zhiheng Huang, Jie Chen, Juanjuan Tang, Wenda Xue, Weiwei Tao, Haoxin Wu, Dawei Wang, Gang Chen

**Affiliations:** ^1^Affiliated Hospital of Integrated Traditional Chinese and Western Medicine, Nanjing University of Chinese Medicine, Nanjing, China; ^2^Center for Translational Systems Biology and Neuroscience and Key Laboratory of Integrative Biomedicine for Brain Diseases, College of Basic Medicine, Nanjing University of Chinese Medicine, Nanjing, China; ^3^Co-innovation Center of Neuroregeneration, Nantong University, Nantong, China; ^4^School of Pharmacy, Nanjing University of Chinese Medicine, Nanjing, China; ^5^Brain Hospital Affiliated to Nanjing Medical University, Nanjing, China; ^6^Interdisciplinary Institute for Personalized Medicine in Brain Disorders, Jinan University, Guangzhou, China

**Keywords:** rapid antidepressant, NMDA receptor, Akt-mTOR signaling, Chaihu-jia-Longgu-Muli-tang, olfactory bulbectomized mice

## Abstract

**Background:** Fast-onset antidepressants are urgently needed. Chaihu-jia-Longgu-Muli-tang (CLM), a classic Chinese herbal medicine, has been used for antidepressant treatment with long history. Olfactory bulbectomization (OB) model is validated for identification of rapid antidepressant efficacy. Here we used OB model for investigating the rapid onset activity of CLM in mice, and also tested the involvement of prefrontal Akt-mTOR and associated AMPA/NMDA receptors as well as hippocampal BDNF in the rapid antidepressant-like effect of CLM.

**Methods:** The OB model was first characterized with depression-like behaviors and the time course changes of the behaviors. The fast onset of antidepressant effect of CLM was evaluated using sucrose preference test, tail suspension test and forced swim test in OB mice after a single administration. The expression of synaptic proteins of AMPA and NMDA subunits as well as Akt/mTOR signaling in the prefrontal cortex, and hippocampal BDNF was evaluated with the immunoblotting method.

**Results:** A single dose of CLM significantly improved the deficiency in the sucrose preference and decreased the immobility time in the tail suspension test in OB mice. In the prefrontal cortex (PFC) in OB mice, there was lower expression level of the AMPA receptor subunit GluR1, rescued by a single dose of CLM. Additionally, the expression of NMDA subunit NR1 was up-regulated in OB mice, whereas mTOR and its upstream Akt signalings were both down-regulated. These deficiencies were reversed by a single dose of CLM. The CLM treatment also attenuated the expressions of NMDA receptor subunits NR2A and NR2B, which did not change in OB mice. In the hippocampus, expressions of GluR1 and brain derived neurotrophic factor (BDNF) were both up-regulated in OB mice, although CLM increased GluR1, but not BDNF.

**Conclusion:** CLM elicited rapid antidepressant-like effects in the OB model mice, and CLM reversal of the abnormality in PFC expression of AMPA and NMDA receptors and associated Akt-mTOR signaling may underlie the effects.

## Highlights

- The olfactory bulbectomization (OB) mice showed abnormal NMDA-Akt-mTOR-AMPA signaling.
- A single dose of CLM reversed the abnormal NMDA-Akt-mTOR-AMPA signaling.
- CLM elicited rapid antidepressant-like effect in OB mice, likely via remedy of NMDA-Akt-mTOR-AMPA signaling.

## Introduction

Major depressive disorder (MDD) is one of the most common diseases of persistent emotion (feeling) stepping down, inhibition of thought and psychomotor retardation. It has been becoming the leading cause of disability and a major contributor to the disease burden of the world’s population (Smith, 2014). Serotonin selective reuptake inhibitors (SSRIs) represent the first-line antidepressants. However, there are several major disadvantages for SSRIs, including the long lag-time of therapeutic action, an appreciate number of non-responsive MDD patients, and adverse reactions (Bull et al., 2002). Therefore, development of fast onset, safe and effective antidepressants is urgently needed.

Recently, ketamine (KET), a N-methyl-D-aspartic acid receptor (NMDAR) antagonist, is found to elicit fast-onset and long-lasting antidepressant effects: a single dose of KET quickly alleviates the depressive symptoms, and the effect may last for several days in both MDD patients and various animal models of depression (Zarate et al., 2006; aan het Rot et al., 2010; Li et al., 2010; Autry et al., 2011; Duman et al., 2012). In the past few years, studies continue to discover the novel mechanisms underlying the rapid antidepressant effects. Emerging evidence support that rapid and persistent improvement of neural plasticity is essential for the process. For example, instant activation of the mammalian target of rapamycin (mTOR) and related signaling pathway is required for the action of KET, whereas the deficiency in this pathway was implicated in depression-like conditions in various chronic animal models (Li et al., 2011; Tang et al., 2015). In the prefrontal cortex (PFC), a single dose of KET stimulates mTOR pathway via activation of Akt or other upstream signaling, leading to increased expression of synaptic proteins, such as α-amino-3-hydroxy-5-methyl-4-isoxazolepropionic acid (AMPA) receptor subunit GluR1, and formation of new spine synapses (Li et al., 2010; Ghasemi et al., 2014). KET also down-regulated expression of NMDA receptor subunit NR1 (Tang et al., 2015). Upregulation of the AMPAR/NMDAR ratio, by increasing AMPAR and/or decreasing NMDAR, is responsible for antidepressant effects and has been shown following acute or chronic KET treatment (Li et al., 2010; Tizabi et al., 2012; Tang et al., 2015). Growing number of studies support that brain derived neurotrophic factor (BDNF) is necessary for mediating antidepressant effects (Tsankova et al., 2006; Martinowich et al., 2007). Experimental studies also suggest that the rapid enhancement of BDNF expression in the hippocampus are responsible for rapid antidepressant-like effect of KET (Li et al., 2010; Autry et al., 2011). The NMDA-Akt-mTOR-AMPA signaling in the PFC and BDNF expression in the hippocampus are probably responsible for the rapid antidepressant-like effect.

Although antidepressant efficacy of KET was well-established experimentally, clinical use of KET is challenged by the potential toxic and addictive effects. Thus, efforts have been made to discover fast-onset and safer antidepressant drugs, including Chinese herbal medicine that has been used clinically in a safe manner. For example, a herbal medicine Yueju pill, has been found to elicit rapid antidepressant-like effect, similar to KET in various animal models in mice (Xue et al., 2013; Tang et al., 2015; Xue et al., 2016). Chaihu-jia-Longgu-Muli-tang (CLM) is also a traditional Chinese herbal formulation in “Shang Han Lun” written by the founding theorist of traditional Chinese medicine, Doctor Zhang Zhongjing, 1800 years ago. The therapeutic effects and mechanisms of CLM on depression and insomnia have been reported previously (Tsujimura et al., 2011; Niitsu et al., 2013). In chronic animal models, the antidepressant effects of CLM following repeated administration linked to reversal of the reduction of dopaminergic and serotonergic transmission, normalization of the dysfunctional hypothalamo-pituitary-adrenal system, and modulation of the glucocorticoid secretion system (Sasaki et al., 1998; Mizoguchi et al., 2003). Clinical observations also suggested a relatively fast antidepressant action of CLM, which, however, has not been scientifically investigated using appropriate animal models.

Olfactory bulbectomization (OB) is a validated model accurately predicting onset time of classical antidepressants or KET-like rapid antidepressant reagents (Ramaker and Dulawa, 2017). After surgically removing bilateral olfactory bulbs, rats or mice demonstrates steady depression-like behaviors, including reduced preference of sucrose, mimicking the core symptom of depression, anhedonia (Redmond et al., 1997; Song and Leonard, 2005). Many neuromolecular features are shared with other depression models, except for increased BDNF expression in the hippocampus in contrast to decreased level in most other models (Angelucci et al., 2004, 2005; Greenwood et al., 2007). It is believed that OB led to dysregulated NMDA-AMPA and Akt-mTOR signaling in PFC similar to other models, which, however, remains to be investigated.

Here, using a mouse OB model, we investigated whether a single dose of CLM was able to elicit rapid antidepressant-like activity; furthermore, we tested the association of NMDA-AMPA receptors and related Akt-mTOR signaling in PFC or increase in hippocampal BDNF with the rapid antidepressant-like action of CLM in OB mice.

## Materials and Methods

### Animals

Kunming mice weighing between 20 and 25 g were purchased from China Academy of Military Medical Sciences (Beijing). Mice aged approximately 6 weeks old and were housed in cages for 7 days before behavioral testing and OB. Animals were maintained in a temperature and humidity-controlled environment, (temperature 22 ± 2°C and room humidity, 50 ± 10%) under a 12: 12 h light/dark cycle. All the mice were offered ad libitum access to food and water. All procedures in this study conformed to the Guide for the Care and Use of Laboratory Animals and were approved by the Institutional Animal Care and Use Committee at Nanjing University of Chinese Medicine.

### Drugs

The medicinal plants used to prepare CLM are Bupleuri radix (Chai Hu), Fossilia ossis mastoid (Long Gu), Scutellariae radix (Huang Qin), Zingiberis rhizoma (Sheng Jiang), Ginseng radix (RenShen), Cinnamomi cortex (Gui Zhi), Hoelen (Fu Ling), Pinelliae tuber (Ban Xia), Radix et Rhizoma Rhei (Da Huang), Osttreae testa (Mu Li), Zizyphi fructus (Da Zao). All the medicinal plants were purchased from Nanjing GuoYi Clinical, Medicinal Material Department (Nanjing, China). CLM was supplied in the form of a water-extracted cream that was manufactured from a mixture of the crude drugs according to a fixed ratio listed in Table 1, and the quality control of different batches of CLM was evaluated using HPLC method (Supplementary Figure S1 and Supplementary Table S1). Briefly, these 11 dried herbal and mineral drugs were soaked in water for 30 min, and then were decocted two times with boiling water in the ratio of 1:8 for 2 h. After collecting all the solution, placed in a water bath at 60°C and evaporated to a target concentration for frozen storage. The yield of the preparation was about 42%. The solutions of the herb preparation and vehicle were administered to mice via intragastric administration at a dosage of 0.1 mL/10 g (body weight), and the concentration of the solutions for administration was 210mg/ml. KET HCl (Gutian Pharmaceuticals, China), dissolved in saline, was administered intraperitoneally.

**Table 1:** Crude drug composition of CLM.

Plant name	Composition (g)	Major components
Bupleuri radix	6	Saikosaponin a, c, d, e
Fossilia ossis mastoid	2.25	Calcium base
Scutellariae radix	2.25	Baicalin, Wogonin
Zingiberis rhizoma	2.25	Gingerol, Shogaol
Ginseng radix	2.25	Ginsenoside
Cinnamomi cortex	2.25	Cinnamic aldehyde
Hoelen	2.25	Eburicoic acid
Pinelliae tuber	3	Homogenistic acid
Radix et Rhizoma Rhei	3	Anthraquinone
Ostreae testa	2.25	Calcium base
Zizyphi fructus	3	Zizyphus saponin, betulinic acid


*These 11 dried herbal and mineral drugs were soaked in water for 30 min, and then were decocted two times with boiling water in the ratio of 1: 8 for 2 h. After collecting all the solution, placed in a water bath at 60°C and evaporated to a target concentration for frozen storage. The yield of the preparation was about 42%. The concentration of the solutions for administration was diluted to 210 mg/ml.*

### Surgery and Treatments

After a 2-week acclimatization period, the animals were randomly divided into two major groups; one group underwent OB and the other were control (CTL). Then, the OB group was divided into three groups, which were vehicle (Veh), CLM and KET. In our previous study, we have screened the dosage of CLM for stable, effective and rapid antidepressant-like effect, and found a dose of 2.1 g/kg was determined to be optimal, which is half equivalent clinical dose. In this study, the CTL and Veh groups were administered with saline intragastrically, CLM group was intragastrically administered with CLM at a dosage of 2.1 g/kg, and KET group was administered intraperitoneally with KET at a dosage of 30 mg/kg (Tang et al., 2015). Olfactory bulbectomy was performed according to the mouse brain anatomy map. Briefly, mice were anesthetized with Chloral hydrate (3.8% 0.1 ml/10 g. To expose the skull, an incision was made in the overlying skin, after which holes were drilled on both sides of the midline (4.5 mm anterior fontanelle; 0.8 mm by the middle line; 2.5–3.0 mm depth). The olfactory bulbs were then bilaterally aspirated using a blunt hypodermic needle (1.0–1.2 cm long with a rounded tip that was 0.80–1.2 mm in diameter) attached to a 10-ml syringe that was used to create suction. Care was taken to avoid damaging the frontal cortex. To stop the bleeding, the holes were filled with swabs and covered with dental cement. 70% alcohol solution was used to eliminate contamination during all of the surgical procedures. The mice were allowed to recover under a warming lamp to help with body temperature maintenance. Each animal was given 14 days to recover from the surgery prior to undergoing any further treatment.

### Behavioral Tests

#### Open Field Test (OFT)

Open field test estimated locomotor activity and anxiety-like behavior. Mice were freely explore for 5 minutes in a well-illuminated (∼300lux) plastic cage (40 cm × 40 cm × 40 cm). A camera was placed in the top of the box for recording the activity. Both the distance traveled (cm) and time spent in central area(s) were analyzed by a computer-based tracking system. Testing apparatus was thoroughly cleaned with 75% ethanol between each test.

#### Sucrose Preference Test (SPT)

The SPT followed a procedure published before with minor modifications. Briefly, mice were housed and exposed to a sucrose solution (2% in tap water) for 72 h, followed by 24 h of water deprivation and mice were offered two individual bottles containing either tap water or 2% sucrose solution for 2 h. Liquid consumption from each bottle was measured by comparing the differences in bottle weight before and after a 2hr exposure. Sucrose preference was measured by the percentage of sucrose solution intake versus total (water + sucrose liquid) intake.

#### Tail Suspension Test (TST)

The apparatus is consisted of 6 chambers which allowed 6 animals to be videotaped and tested at one time. In a chamber which is isolated acoustically and visually, an individual mouse was individually suspended 1cm from the tip of the tail to the vertical bar with adhesive tape. A camera positioned in front of the TST box was used to record the animal’s behavior for 6 min. Total immobility time during the last 4 min was analyzed by ANY-maze software. No animal climbed its tail during the test.

#### Western Blot

After the mouse behavioral test was finished, the brain was taken on ice. The entire hippocampus and PFC were lysed in RIPA buffer containing protease inhibitors and phosphatase inhibitors. Protein concentration was determined colorimetrically by BCA assay (Pierce, Rockford, IL, United States). Protein lysates were separated by SDS-PAGE electrophoresis and were transferred onto polyvinylidene difluoride (PVDF) membranes. After blocking with 1% BSA for 1 h, the membranes were incubated with primary antibodies. BDNF (Santa Cruz Biotechnology, sc-546, 1 : 400), GluR1(Cell Signaling Technology, #13185, 1:1000), NMDAR1 (Cell Signaling Technology, 5104s, 1 : 1000), NR2A (Cell Signaling Technology, #4205, 1 : 1000), NR2B (Cell Signaling Technology, 4212s, 1 : 1000), p-mTOR (Cell Signaling Technology, #2971, 1:1000), mTOR (Cell Signaling Technology, #2972, 1:1000), phosphor-Akt (Cell Signaling Technology, #4060, 1:1000), Akt (Cell Signaling Technology, #9272, 1: 1000), and tubulin (Proteintech, 10094-1-AP, 1 : 2000) were used at 4°C overnight. The next day, blots were washed three times in TBST and incubated with horseradish peroxidase conjugated anti-mouse or anti-rabbit secondary antibody (1:3000) for 1 h. After final three washes with TBST, the bands were visualized using the Super Signal West Pico Chemiluminescent Substrate (Thermo Fisher Scientific Inc.). BDNF, mTOR, Akt, NR1, NR2A, NR2B and GluR1 were all normalized to β-Tubulin bands, and pro-BDNF, mature BDNF, p-mTOR, p-Akt bands were normalized to total protein levels then expressed as a percentage. All experiments were performed 3 times.

### Statistical Analysis

All data are presented as means ± SEM. Two-sample comparisons were carried out using two-tailed Student’s *t*-test. Others was performed by one-way analysis of variance (ANOVA) followed by the Bonferroni multiple comparison tests. A value of *p* < 0.05 was considered statistically significant.

## Results

### A Single Dose of CLM Elicited Rapid Antidepressant-Like Activity in OB Mice

**FIGURE 1:**
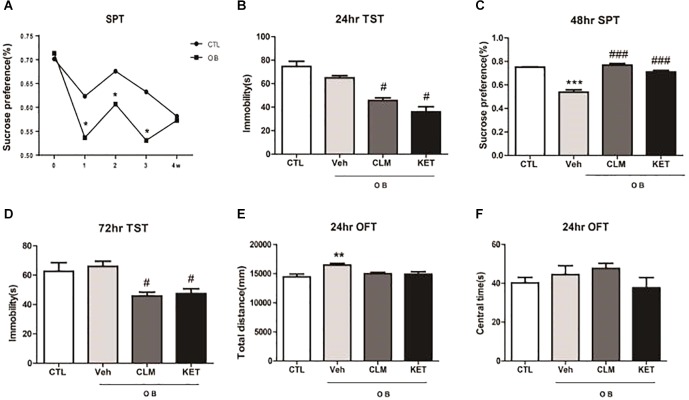
Behavioral effects at different time points after a single ketamine and CLM treatment on OB mice. Control animals (CTL) received vehicle treatment, and animals exposed to olfactory bulbectomy received a single administration of vehicle (OB), Chaihu-jia Longgu-Muli decoction (CLM) or ketamine (KET). **(A)** Sucrose preference test at 1 week, 2 weeks, 3 weeks, and 4 weeks after olfactory bulbectomy surgery (*T*-test, ^∗^*p* < 0.05 and *n* = 6–10/group). **(B)** TST (ANOVA, *F*(3,30) = 25.85, *p* < 0.0001, and *n* = 7–8/group) was tested at 24 h. **(C)** SPT (ANOVA, *F*(3,35) = 57, *p* < 0.0001, and *n* = 9/group) was tested at 48 h. **(D)** TST (ANOVA, *F*(3,32) = 4.735, *p* < 0.01, and *n* = 8–9/group) was tested at 72 h. ^∗^*p* < 0.05, ^∗∗^*p* < 0.01, ^∗∗∗^*p* < 0.001, compared to CTL; ^#^*p* < 0.05, ^##^*p* < 0.01, ^###^*p* < 0.001, compared to Veh. **(E,F)** OFT was performed 24 h post administration of CLM, KET or saline [*F*(3,23) = 5.591, *p* < 0.05 for total distance, ANOVA, *F*(3,23) = 1.225, *p* > 0.05 for central time, and *n* = 6/group].

Following the surgery for OB, SPT was performed weekly for 4 weeks to monitor the time course of depression-like response (Figure 1A). OB resulted in a significant decrease in sucrose preference at week 1, 2, and 3 (*p* < 0.05 vs. CTL). By week 4 post surgery, OB group no longer differed from the control group (*p* > 0.05), indicating that the OB surgery induced a relatively long but reversible depression-like behavior. Thus, in the following experiments, animals were treated at week 2 post surgery with a single dose of CLM or ketamine (KET). Then, different behavioral tests were performed at 24, 48, and 72 h post administration. Although the immobility time in OB mice did not differ from the non-OB control group, a single administration of CLM and KET both significantly decreased the immobility time in TST in OB mice at 24 hr, compared to vehicle control (*p* < 0.01, Figure 1B) and at 72 h (*p* < 0.05, Figure 1D). CLM or KET increased sucrose preference in SPT (*p* < 0.001, Figure 1C) post a single administration. By 2 weeks after surgery, mice showed increased locomotor activity (*p* < 0.05, Figure 1E) in the OFT. Administration of CLM or KET did not affect the time spent in central area or total distance in the OFT (Figures 1E,F).

### CLM Normalized AMPA and NMDA Receptor Expression in PFC of OB Mice

**FIGURE 2:**
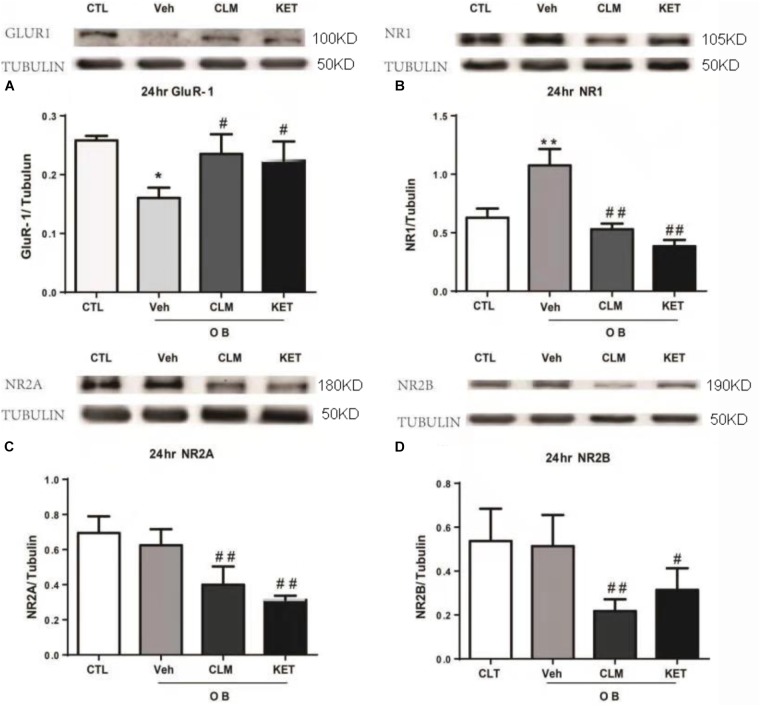
Western blotting detection of expressions of Glur1 and NMDA receptor subunits in the PFC of OB mice at 24 h post a single administration of CLM or KET. **(A)** GluR1, ANOVA, *F*(3,23) = 16.43. **(B)** NR1, ANOVA, *F*(3,23) = 68.82. **(C)** NR2A, ANOVA, *F*(3,15) = 18.15. **(D)** NR2B, ANOVA, *F*(3,19) = 8.797; Data are means ± SEM, and *n* = 4–6/group. ^∗^*p* < 0.05, ^∗∗^*p* < 0.01, compared to CTL; ^#^*p* < 0.05, ^##^*p* < 0.01, compared to Veh.

The expression of NMDA and AMPA receptor subunits in PFC were examined across groups at 24 h post a single dose of CLM or ketamine. Compared to normal group, there was significant reduction in the AMPA receptor subunit GluR1 expression (*p* < 0.05), which were restored following a single dose of CLM (*p* < 0.05) and KET (*p* < 0.01, Figure 2A). The OB mice also showed increased level of NMDA receptor subunit NR1 expression (*p* < 0.05, Figure 2B), which was suppressed by a single dose of CLM (*p* < 0.01), or ketamine (*p* < 0.01), Figure 2B). Although OB did not change the expression of NR2A or NR2B, CLM or ketamine decreased expression of NR2A subunit (*p* < 0.01, Figure 2C) and NR2B subunit expression (*p* < 0.01 for CLM, *p* < 0.05 for KET Figure 2D) in OB mice.

### CLM Activited AKT-mTOR Signaling in the PFC

**FIGURE 3:**
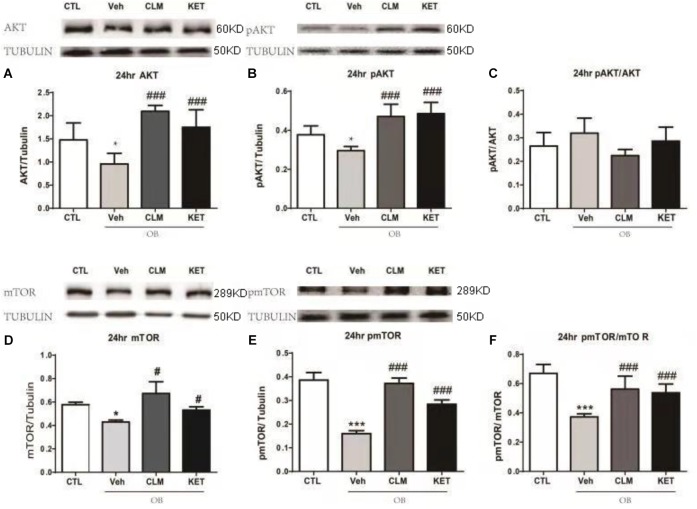
Western blotting detection of expressions of AKT and mTOR signaling in the PFC of OB mice at 24 h post a single administration of CLM or KET. **(A)** AKT, ANOVA, *F*(3,23) = 15.91, *p* < 0.0001. **(B)** pAKT, ANOVA, *F*(3,23) = 19.15, *p* < 0.0001. **(C)** pAKT/AKT, *F*(3,23) = 3.331, *p* < 0.05. **(D)** mTOR, ANOVA, *F*(3,19) = 17.62, *p* < 0.0001. **(E)** pmTOR, ANOVA, *F*(3,19) = 106.5, *p* < 0.0001. **(F)** pmTOR/mTOR, *F*(3,19) = 19.34, *p* < 0.0001. Data are means ± SEM, and *n* = 5–6/group. ^∗^*p* < 0.05, ^∗∗^*p* < 0.01, ^∗∗∗^*p* < 0.001, compared to CTL; ^#^*p* < 0.05, ^##^*p* < 0.01, ^###^*p* < 0.001 compared to Veh.

In the PFC, OB mice showed significantly lower level of total Akt and pAKT (*p* < 0.05, Figures 3A–C), without change in the ratio of pAkt to Akt. A single dose of CLM or KET improved the deficits in total Akt and pAKT, without effect on pAkt/Akt. Furthermore, total mTOR, pmTOR as well as pmTOR/mTOR were all reduced in the OB mice (*p* < 0.05, Figures 3D–F), which were reversed by a single dose of CLM (*p* < 0.001), similar to KET (*p* < 0.001, Figures 3D–F).

### CLM Increased GluR1 but Not BDNF in Hippocampus in OB Mice

**FIGURE 4:**
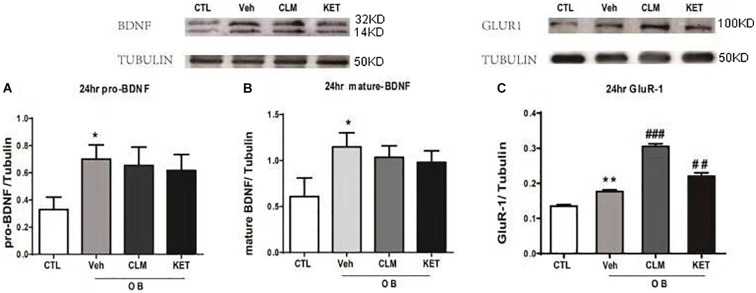
Western blotting detection of expressions of BDNF and GluR1 in the hippocampus in OB mice at 24 h post a single administration of CLM or KET. **(A)** pro-BDNF, ANOVA, *F*(3,19) = 7.243, *p* < 0.01. **(B)** mature-BDNF, ANOVA, *F*(3,23) = 13.5, *p* < 0.001. **(C)** GluR1, ANOVA, *F*(3,23) = 101.8, *p* < 0.0001. Data are means ± SEM, and *n* = 5–6/group. ^∗^*p* < 0.05, ^∗∗^*p* < 0.01, compared to CTL; ^##^*p* < 0.01, ^###^*p* < 0.001 compared to Veh.

The rapid enhancement of BDNF is also a neuroplastic mechanism of rapid antidepressant effect. Interestingly, the expression of BDNF and GluR1 was up-regulated at 24 hr in the veh group, compared to the CTL group (*p* < 0.05 Figures 4A,B, *p* < 0.01 Figure 4C). Both CLM and KET group increased the GluR1 expression (*p* < 0.001 for CLM, *p* < 0.01 for KET, Figure 4C), but they didn’t have any difference in mature and pro-BDNF expression compared to Veh (*p* > 0.05, Figures 4A,B).

## Discussion

We aimed to test a traditional Chinese herbal medicine CLM for a rapid antidepressant activity and to examine associated neuromolecular substrate. We found a single dose of CLM instantly induced an antidepressant-like action, which lasted for 3 days. Using the OB depression model in mice, we found the deficits in sucrose preference in OB mice, which was reversed by a single dose of CLM, similar to ketamine. Moreover, in the PFC, mice with OB showed decreased level of expression of GluR1 and increased level of NR1, which was reversed by a single dose of CLM. There was also remarked deficiency in Akt and mTOR signaling regulating neural plasticity in OB mice, which was ameliorated by a single dose of CLM. These results suggested that normalization of NMDA and AMPA receptors and related Akt-mTOR signaling may account for the rapid antidepressant-like activity of CLM.

### Rapid Antidepressant-Like Effect of CLM in OB Mice

In OB model, a period of 2 weeks is suggested to be optimal for the development of the bulbectomy syndrome, including hyperactivity and alterations in exploration and social behavior (van Riezen and Leonard, 1990). Consistent with these results, we found OB mice were hyperactive in OFT at post surgery week 2, and displayed a robust depression-like phenotype on the basis of the performance in SPT at week 1, but it was reversible by week 4. In this model, it requires 14 days for SSRIs to improve depressive behaviors, recapitulating a minimum of 2–4 weeks of continuous treatment for onset of antidepressant effects of SSRIs in humans (Redmond et al., 1997). The present findings showed that, similar to ketamine, only a single dose of CLM was capable to reverse the deficits in SPT, and the effect lasted for 3 days. Additionally, the immediate and persistent antidepressant effect of CLM was also evident in the test of TST in both OB model and non-OB conditions. As OB chronic depression model is one of the best to be used for testing the rapid antidepressant potential after a single or a few number of dosing (Ramaker and Dulawa, 2017), the results from this study suggest the fast-onset antidepressant-like effect of CLM.

### Akt-mTOR Signaling Pathway Was Involved in the Rapid Antidepressant Effect of CLM

We found the level of phosphorylation of AKT and mTOR decreased in PFC of OB mice, which was increased post a single administration of CLM. Akt-mTOR signaling is important for neural plasticity for rapid antidepressant effects. The activation of Akt-mTOR signaling is responsible for persistent increase of expression of synaptic proteins such as GluR1 (Li et al., 2010). In the current study, we found for the first time that the phosphorylation of Akt and mTOR as well as the expression of GluR1 decreased in PFC of OB mice, consistent with the results in the chronic mild stress model and the postpartum depression model (Li et al., 2011; Tang et al., 2015; Xia et al., 2016). A single administration of CLM rescued the phosphorylated Akt, phosphorylated mTOR and expression of synaptic protein GluR1, similar to ketamine. It is worth noting that, the expression of total mTOR or total Akt did not alter in other chronic depression models, but it was down-regulated in OB mice, rescued by a single dose of CLM here. These results may indicate some distinct molecular deficits in the OB model (Chandran et al., 2013). Nonetheless, these molecular deficits were still repairable by a single dose of CLM or ketamine.

### The Regulation of Synapse Protein Related to CLM’s Rapid Antidepressant Activity

The current study showed that, contrary to GluR1, NR1 was overexpressed in PFC of OB mice, which was reversed by CLM and ketamine. The expression of NR1 in the PFC or hippocampus was up-regulated in mice of chronic mild stress, learned helplessness and pre-pregnancy stress depression models (Autry et al., 2011; Tang et al., 2015; Xia et al., 2016), and here we provided consistent evidence in OB mouse model, supporting the association of upregulated NR1 expression with depression-like behavior. Additionally, the expression of NR2A and NR2B subunits were also evident in OB mice, which normally were not observed in other models. A single dose of CLM or ketamine decreased all of the expression level of NMDA subunits, demonstrating that CLM and ketamine exerted overall inhibitory effect on the NMDA receptor. Similar to ketamine and Yueju pill, a single dose of CLM reversed both the decrease of AMPA receptor GluR1 and increase of NMDA receptor in OB mice, thus enlarged the ratio of AMPA/NMDA in the PFC (Andres et al., 2013; Tang et al., 2015). Interestingly, in the hippocampus of OB mice, the expression of GluR1 was increased, but not decreased. The expression of GluR1 in OB mice was further enhanced by CLM or ketamine stably. On the other hand, BDNF was significantly increased in hippocampus of OB mice, consistent with previous reports (Hellweg et al., 2007). However, the hippocampal BDNF level in OB mice failed to alter by a single dose of CLM. These observations suggest that rapid normalization of the ratio of AMPA to NMDA receptors may represent an important mechanisms underlying the rapid reverse of depression-like behavior in OB mice by CLM.

## Conclusion

The present study demonstrated the fast-onset antidepressant-like activity of CLM. An equivalent to half of clinical dose of CLM elicited an antidepressant effect on OB mice post an acute administration and the effect lasted for 3 days. In OB mice, a single dose of CLM rescued the deficient Akt-mTOR signaling which controls the expression of synaptic proteins including GluR1. Additionally, the dysregulations of the subunits of NMDA was also reversed by CLM. These findings suggest that the activation of Akt-mTOR signaling and normalization of AMPAR/NMDAR ratio in the PFC may underlie the rapid antidepressant-like effect of CLM in OB mice.

## Author Contributions

XW and GC conceived and designed the experiments. XW, ZZ, QS, ZH, WX, WT, and JT performed the experiments. JC, ZH, and DW analyzed the data. XW, ZZ, ZH, JC, JT, HW, DW, and GC contributed to the writing of the manuscript.

## Conflict of Interest Statement

The authors declare that the research was conducted in the absence of any commercial or financial relationships that could be construed as a potential conflict of interest.

## Abbreviations

AMPA: α-amino-3-hydroxy-5-methyl-4-isoxazolepropionic acid
BDNF: brain derived neurotrophic factor
CLM: Chaihu-jia-Longgu-Muli-tang
KET: Ketamine
MDD: major depressive disorder
mTOR: mammalian target of rapamycin
NMDA: N-methyl-D-aspartate
OB: olfactory bulbectomization
OFT: open field test
PFC: prefrontal cortex
SPT: sucrose preference test
SSRIs: serotonin selective reuptake inhibitors
TST: tail suspension test
