# Supplementary material for: Involvement of NMDA-AKT-mTOR Signaling in Rapid Antidepressant-Like Activity of Chaihu-jia-Longgu-Muli-tang on Olfactory Bulbectomized Mice
Source: Front Pharmacol. 2019 Jan 9;9:1537. doi: 10.3389/fphar.2018.01537 (PMC6333740; doi:10.3389/fphar.2018.01537)
Supplement: Supplementary file 2 [file Data_Sheet_1.PDF]

Supplemental material:

Figure legend

S1: The quality control of different batches of CLM evaluated by HPLC. Mobile phase: 0.5% Phosphoric acid solution (A) and Acetonitrile (B); centrifuge at 100,00 rpm for 5 minutes. Gradient elution: 0-90 min, 90-10% A, 10-90% B; 90-92 min, 10-0% A, 90-100% B. The flow rate was 1ml/min, working potential was 650 mV for the guard cell and 300 mV (E1) and-150 mV (E2) for the analytical cell and the column temperature was ambient. The injection volume was 10 μL, and the column temperature was maintained at 35℃. The DAD detector was set at 260 nm for acquiring chromatograms. Baiclin in CLM was tested by HPLC and used as a reference. Colors represent separated groups of herbs. The X axis represents acquisition time (Min) and the Y axis indicates intensity (AU). As shown in S1, there was a similar chromatographic pattern in different batches of CLM herbal solutions. This suggested a good reproducible for this herbal medicine preparation.

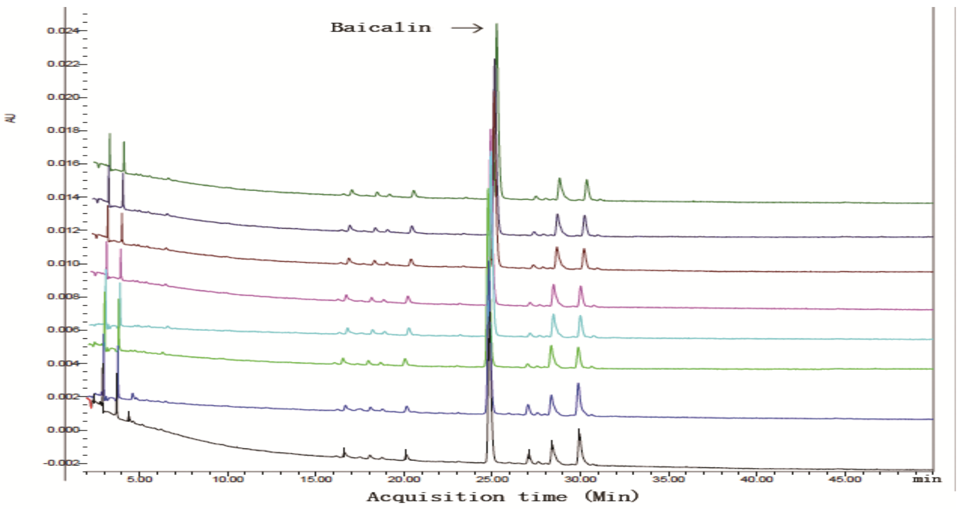

Table 1: Both peak area and concentration of baicalin in CLM by HPLC analysis.

Sample	Concentration (mg/ml)	Peak area (AU)
Baicalin	0.028	103±1
CLM	0.086	125±1
